# Radial Scar: a management dilemma

**DOI:** 10.1007/s11547-021-01344-w

**Published:** 2021-03-20

**Authors:** Charlotte Marguerite Lucille Trombadori, Anna D’Angelo, Francesca Ferrara, Angela Santoro, Paolo Belli, Riccardo Manfredi

**Affiliations:** 1 Università Cattolica del Sacro Cuore, Dipartimento Universitario di Scienze Radiologiche ed Ematologiche, Largo Francesco Vito 1, 00168 Rome, Italy; 2 UOC Radiologia Generale ed Interventistica Generale, Area Diagnostica per Immagini, Dipartimento Diagnostica per Immagini, Radioterapia Oncologica ed Ematologia, Fondazione Policlinico Universitario A. Gemelli IRCCS, Rome, Italy; 3 UOC di Gineco-patologia e Patologia Mammaria, Dipartimento per la Salute della Donna e del Bambino e della Salute Pubblica, Fondazione Policlinico Universitario A. Gemelli IRCCS, Rome, Italy

**Keywords:** Radial scar, B3-lesions, Vacuum-assisted biopsy, Radial scar management

## Abstract

Radial scar (RS) or complex sclerosing lesions (CSL) if > 10 mm is a benign lesion with an increasing incidence of diagnosis (ranging from 0.6 to 3.7%) and represents a challenge both for radiologists and for pathologists. The digital mammography and digital breast tomosynthesis appearances of RS are well documented, according to the literature. On ultrasound, variable aspects can be detected. Magnetic resonance imaging contribution to differential diagnosis with carcinoma is growing. As for the management, a vacuum-assisted biopsy (VAB) with large core is recommended after a percutaneous diagnosis of RS due to potential sampling error. According to the recent International Consensus Conference, a RS/CSL lesion, which is visible on imaging, should undergo therapeutic excision with VAB. Thereafter, surveillance is justified. The aim of this review is to provide a practical guide for the recognition of RS on imaging, illustrating radiological findings according to the most recent literature, and to delineate the management strategies that follow.

## Background

Radial scar (RS) is a benign breast lesion classified with the B-coding system as a lesion with uncertain malignant potential (B3-lesion) [1].

**Fig. 1:**
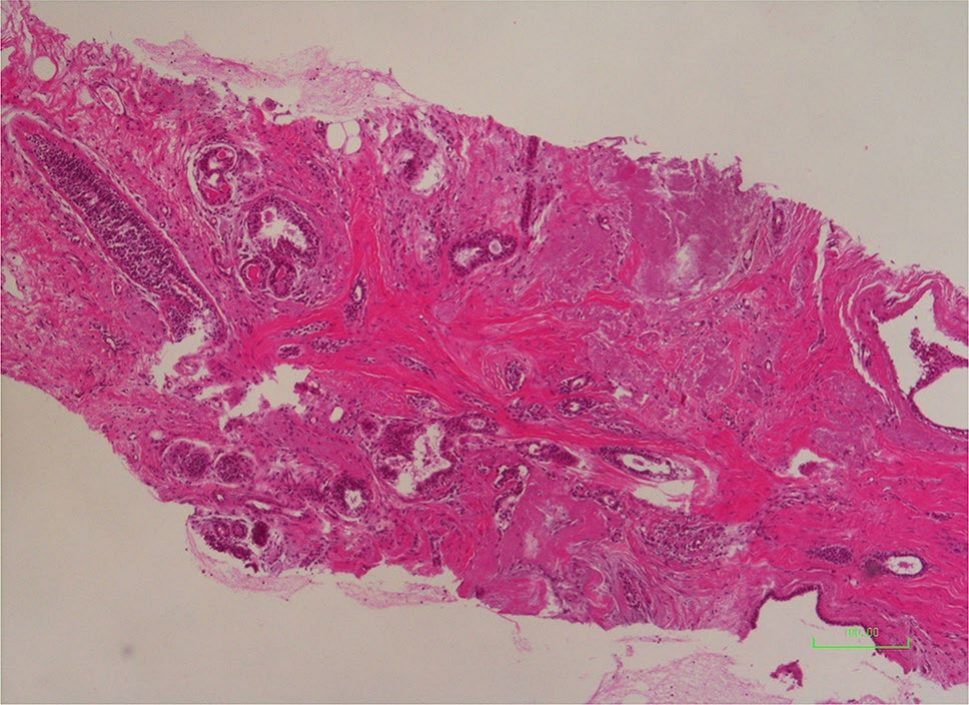
Surgical specimen shows typical aspect of RS: lesion with stellate architecture, prominent fibroelastosis with basophilic elastic material, obliterated ducts, compressed tubular structures with two cell layers (including myoepithelium, CK14 +) and hyalinized stroma. (Hematoxylin–eosin stain [H&E]; magnification × 4)

It is histologically characterized by a central area mimicking a scar, containing one to several ducts showing obliterative mastopathy and surrounded by elastic fibers. In addition, other ducts converge into the scar-like area in a stellate fashion [2, 3] (Fig. 1). When larger than 10 mm, a lesion presenting these features is called complex sclerosing lesion (CSL).

Etiology of RS remains obscure, although several theories have been proposed.

Some authors [4] suggested it may begin as a reaction to an unknown injury, that heals with focal areas of fibrosis and elastosis contracting centrally and forming the characteristic stellate appearance.

Battersby and Anderson [5] suggested a role for chronic inflammation and demonstrated that RS is a lesion characterized by the presence of central myofibroblast activity, parenchymal degeneration and sclerosis, characterized by an early stage with a prevalence of myofibroblasts and a late stage with more prominent elastosis and fewer myofibroblasts.

Other authors postulated that RS [6] arises as a manifestation of fibrocystic changes, considering that the frequency of RS is higher among women with fibrocystic disease.

RS is seen more frequently in women 30–60 years old [7] and it is generally clinically occult and often not palpable regardless of size and superficiality within the breast [8].

It is usually diagnosed at image-guided biopsy and has an incidence ranging from 0.6 to 3.7% that is growing in the last years due to the introduction of population-based screening programs and the increasing use of digital breast tomosynthesis (DBT) [9].

Considering the mammographic frequency, Tabar and Dean found a prevalence of 0.9 radial scars every 1000, in screening examinations [10].

In autopsy series, the incidence of RS has ranged from 14 to 28% depending on the frequency of the section sampling method, since it is not rare for a breast to contain multiple RS that are often millimetric.

RS represents a trick for breast radiologists, because of its morphologic similarity with malignancy resulting in a difficult differential diagnosis, and for the pathologists, because of its association with other proliferative lesions and the possibility of founding foci of intraductal or invasive carcinoma within or adjacent to the lesion. Hence, the importance of the diagnosis and management remains controversial.

The aim of this review is to provide a practical guide for the recognition of RS on imaging, illustrating radiological findings according to the most recent literature and delineate the management strategies that follows.

## Imaging findings

Imaging is crucial for diagnosis of RS, in some cases, found occasionally during routine radiological screening. In the last years, the role of DBT as a screening and diagnostic tool has been demonstrated to help the radiologist detecting mammographic architectural distortions, resulting in an increasing incidence of both carcinoma and RS [11–13].

The use of magnetic resonance imaging (MRI) in diagnosis or in evaluation of RS is still controversial; it may be used as a problem-solving tool for inconclusive clinical or mammographic findings, or to rule out malignancy in patients diagnosed with RS after core needle biopsy (CNB) resulting in a valuable help for management assessment [14].

The main imaging findings are resumed in Table 1.

**Table 1:** RS/CSL imaging findings

DM/DBT	US	MRI
“Black Star”: central radiolucency radiating long, thin spicules	Irregularly shaped hypoechoic mass/distorted parenchymal area: ill-defined borders	Stellate architectural distortion: no mass effect mild or no enhancement
“White Star”: stellate opacity	Round or oval mass: circumscribed margins	Irregular and spiculated “tumor-like” mass
Group of microcalcifications	Focal area of shadowing with no discernible mass	Oval or round mass: smooth margins
	Not visible	Mass or architectural distortion without enhancement

## Digital mammography (DM) and digital breast tomosynthesis (DBT)

**Fig. 2:**
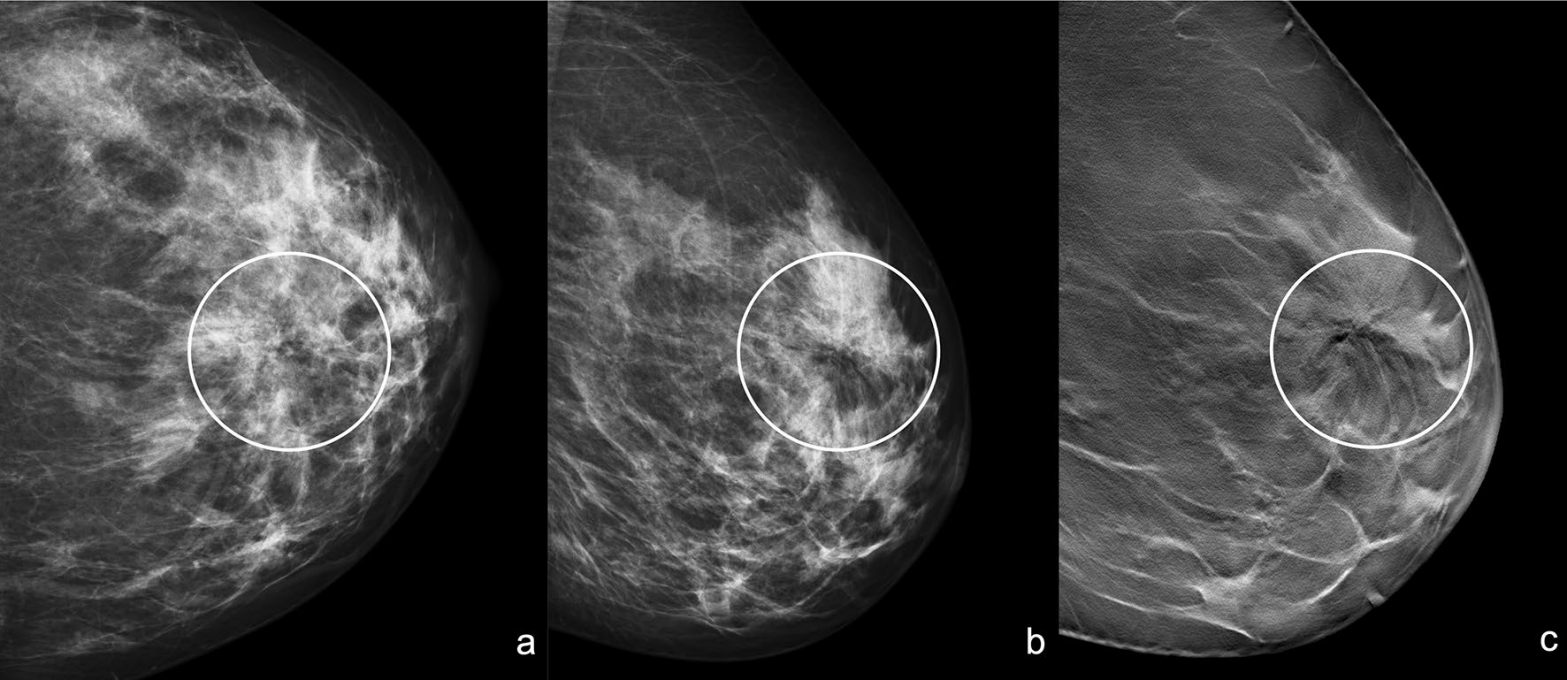
“Black Star”: Left craniocaudal (**a**) and mediolateral oblique (**b**) mammograms show an area of architectural distortion with radiolucent core in the union of upper quadrants (white circle). Left mediolateral oblique tomosynthesis (**c**) confirms the area of architectural distortion and shows better the radiolucent core with the radiating long thin spicules (white circle)

**Fig. 3:**
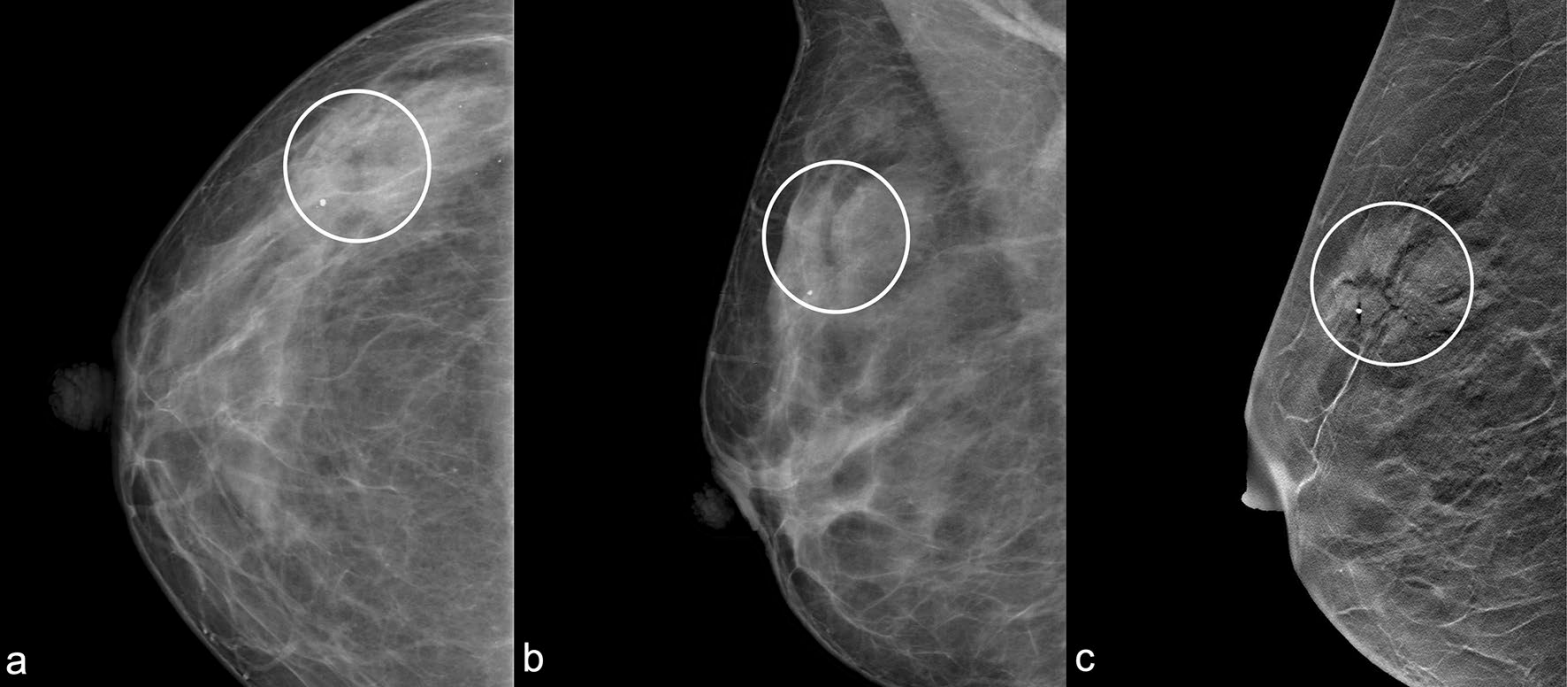
“Black Star”: Right craniocaudal (**a**) and mediolateral oblique (**b**) mammograms show an area of architectural distortion with radiolucent core in the upper-outer quadrant (white circle). Right mediolateral oblique tomosynthesis (**c**) shows better the architectural distortion and the radiolucent core (white circle)

The most typical appearance on digital mammography (DM) and on DBT of RS is the architectural distortion, the “black star” (Fig. 2 and 3), described by Tabar and Dean [10] including five criteria:

-Central radiolucency;
-Radiating long, thin spicules;
-Varying appearance in different projection;
-Radiolucent linear structures parallel to the spicules;
-Absence of palpable lesion/skin changes.

The presence of radiolucent core does not exclude malignancy; in fact, it is challenging to differentiate the central radiolucent core from superimposed background fat [15].

**Fig. 4:**
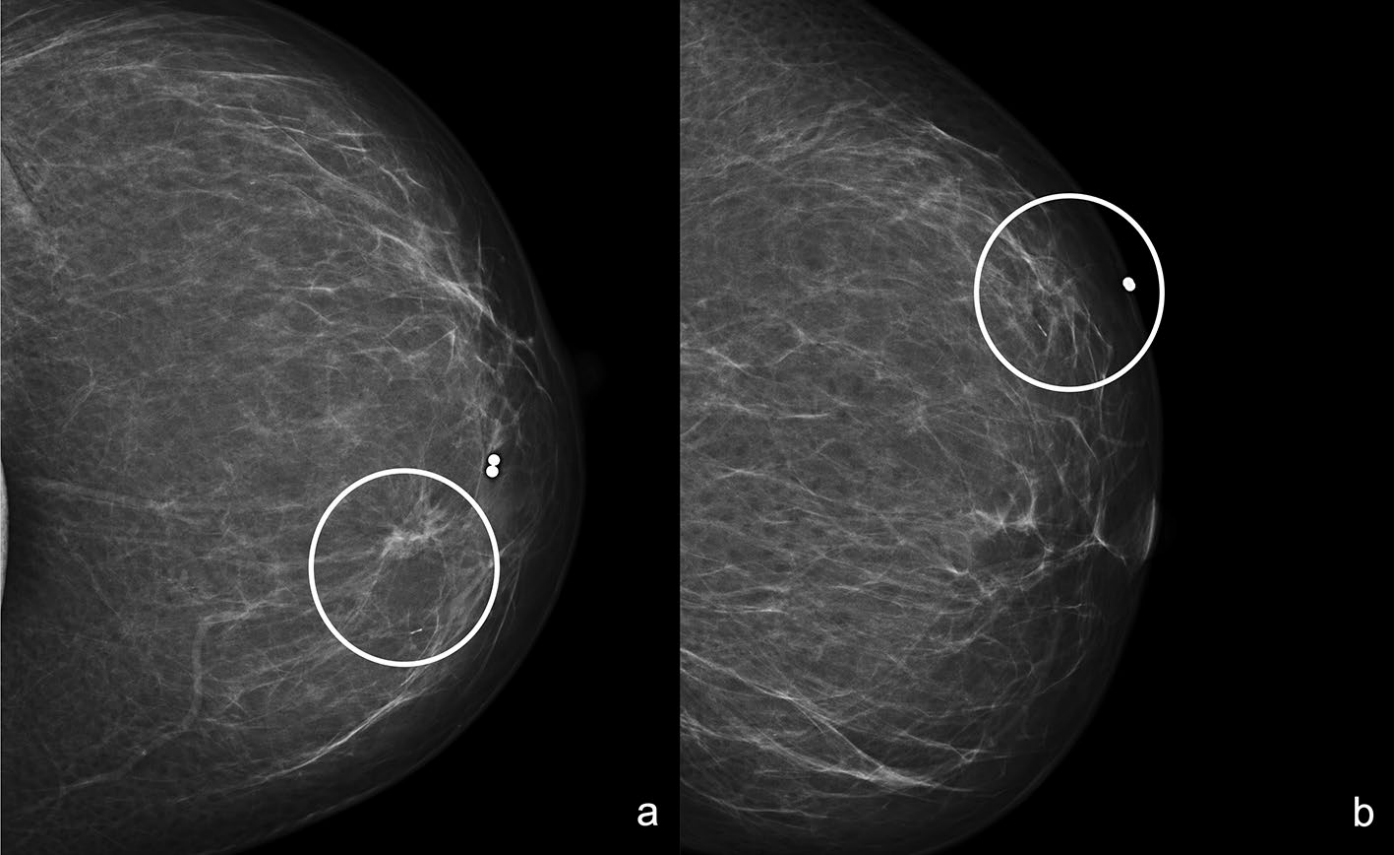
“White Star”: Left craniocaudal (**a**) and mediolateral oblique (**b**) mammograms reveal a stellate opacity with ill-defined borders and spiked linear extensions (white circle) in the upper- inner quadrant. Radiopaque metallic landmark was positioned before surgery

RS can also appear as a stellate opacity (the “white star”) (Fig. 4) that is a mass having irregular borders and spiked linear extensions, which lead out toward adjacent tissue; the morphology is similar to carcinoma and differential diagnosis is even more difficult.

Cohen et al. [7] described how several studies tried to retrospectively identify cases of spiculated masses with features suggesting that the lesion excised was a RS [15–17]. At surgery, 17–59% of lesions were misclassified, particularly because of the misleading presence of radiolucent centers. This finding emphasizes the struggle of differentiating RS and carcinoma at DM.

Some studies suggested that length of spicules of a spiculated lesion contributes to differential diagnosis of RS versus carcinoma: the longer the spicules are compared to the lesion diameter, the more likely the stellate lesion is benign [18, 19]; more specifically, considering (D) the diameter of the stellate lesion including spicules and (d) the lesion diameter, Hagay [20] stated that a D/d ratio higher than two suggests benignancy.

**Fig. 5:**
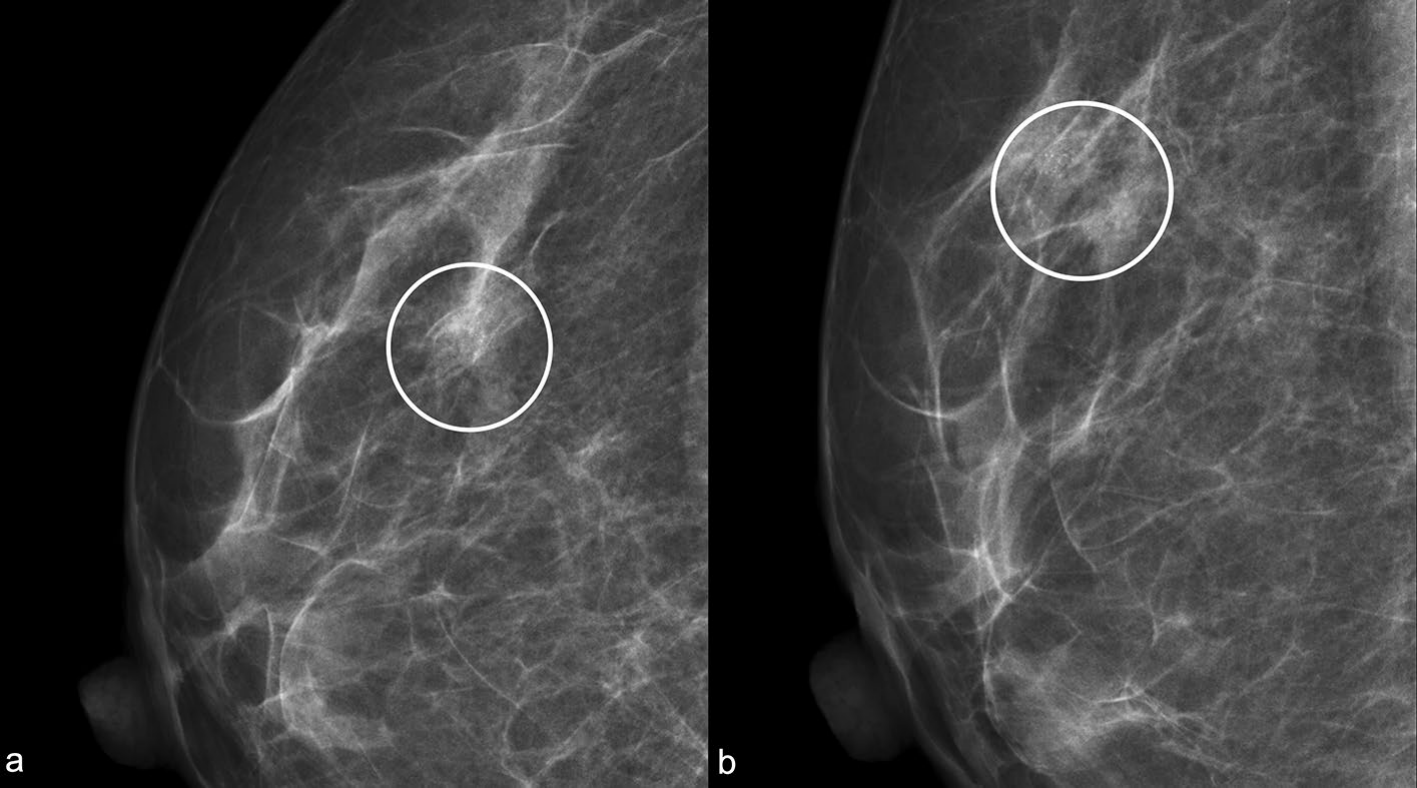
Right craniocaudal (**a**) and mediolateral oblique (**b**) mammograms show an area of microcalcifications with in the upper-outer quadrant (white circle)

Rarely, RS appears on DM like a group of microcalcifications (Fig. 5). Calcifications are often related to the benign proliferative fibrocystic changes and sclerosing adenosis that coexist within and around those lesions. Anyway, morphologic characteristics of these calcifications are often non-specific resulting inadequate to differentiate benign from malignant disease [7]. Miller CL et al. [21] reported that the radiological appearances of a mass or architectural distortion on DM or ultrasound (US) are more likely to be upgraded to carcinoma compared with RS’s presenting as calcifications.

Numerous studies have demonstrated that DBT increases detection of RS [22, 23], mostly in recognizing and defining tomographic characteristics of benign architectural distortion like symmetric spiculation with spoke-wheel morphology and central-lucency on mammographic imaging [12, 13]. Nevertheless, there are still no DBT-specific features to allow a certain differentiation of RS from cancer.

## Ultrasound (US)

On US, RS can have variable aspects. It is not always sonographically visible, and it is demonstrated that finding an architectural distortion without correlative findings on US was less likely to represent malignancy than architectural distortion with correlative sonographic findings [12, 24]; nevertheless, an architectural distortion on DM with no US findings needs further investigation with stereo-biopsy.

**Fig. 6:**
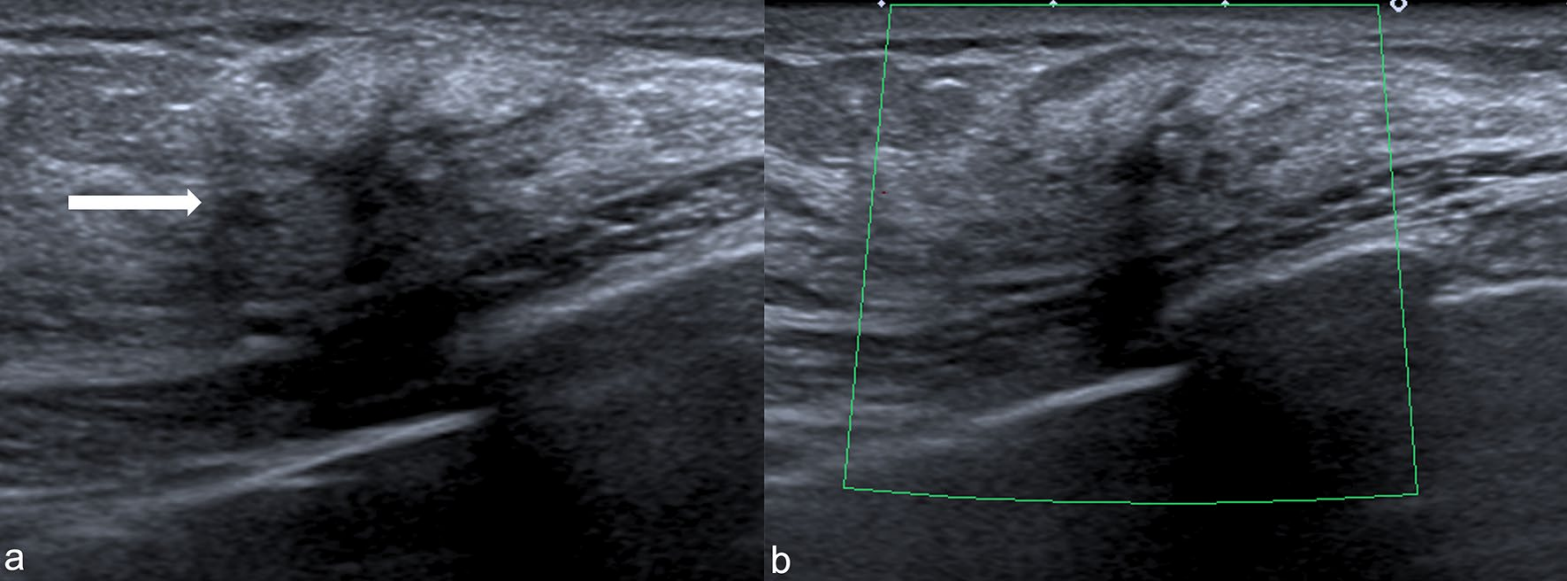
US demonstrates an irregularly shaped hypoechoic distorted parenchymal area, showing ill-defined borders, with posterior acoustic shadowing (white arrow)

**Fig. 7:**
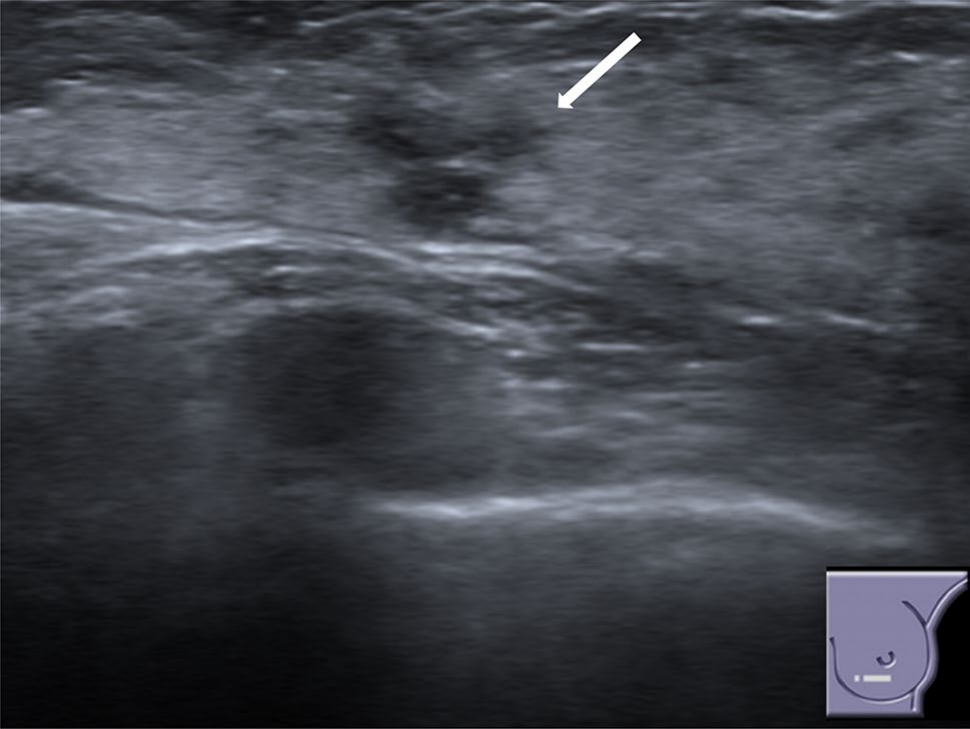
US shows a mass with circumscribed margins without posterior acoustic shadowing (white arrow)

**Fig. 8:**
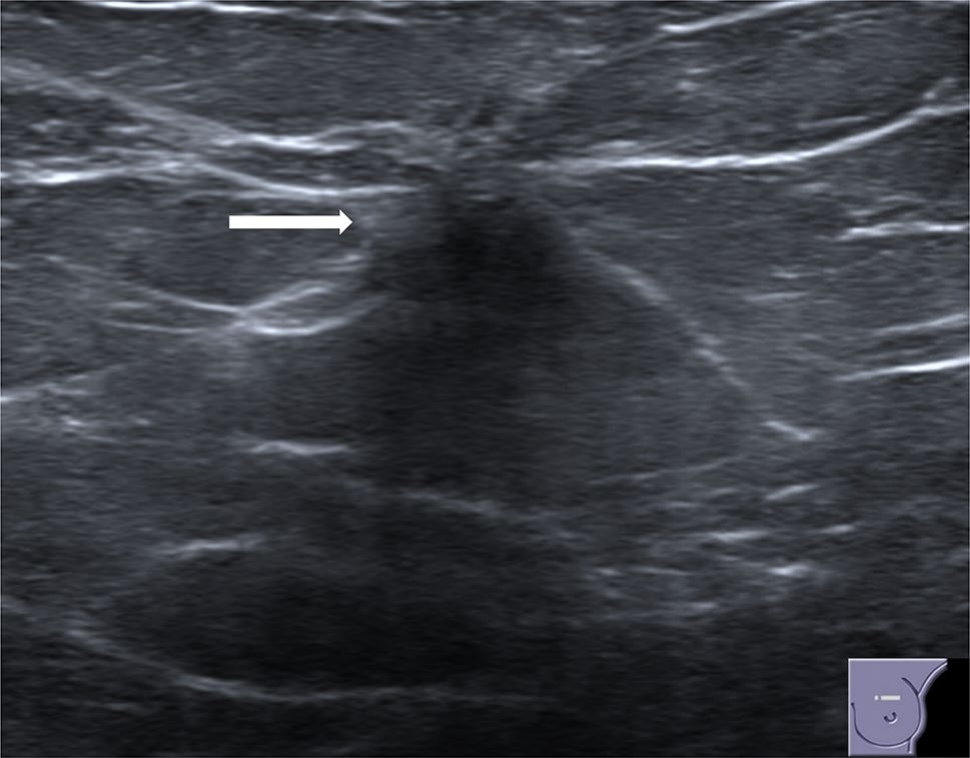
US shows a focal area of shadowing with no discernible mass (white arrow)

When visible, RS can appear as [7]:

-Irregularly shaped hypoechoic mass or distorted parenchymal area, showing ill-defined borders, with or without posterior acoustic shadowing, virtually identical to a carcinoma of the breast (Fig. 6);
-Round or oval mass with circumscribed margins and without posterior acoustic enhancement or shadowing (Fig. 7);
-Focal area of shadowing with no discernible mass (Fig. 8).

Cawson et al. [25] have defined US findings that suggest the likelihood of RS instead of cancer:

-Absence of echogenic halo;
-Presence of tiny sonographic cysts;
-Absence of shadowing and breast architecture disruption.

The introduction of breast elastography was investigated in the literature [26–28], suggesting that frequently RS has an inherent stiffness comparable to that of invasive breast cancer, leading to false-positive elastography results. For this reason, breast elastography does not appear reliable for differentiating RS from malignant lesions.

In a recent study, Vourtsis and Kachulis [29] evaluated the use of automated breast ultrasound (ABUS) compared to conventional hand-held US (HHUS) in the visualization and characterization of breast lesions. The authors showed that ABUS confers an added value on the coronal plane, helping in recognition of architectural distortion. Particularly, ABUS allows the detection of RS that was not recognized at DM or HHUS.

Anyway, RS can’t be reliably differentiated from malignancy on the basis of DM/DBT features alone, correlation with US is fundamental and biopsy is always recommended.

## Magnetic resonance imaging (MRI)

The use of MRI in breast imaging has progressively increased over last decades and its capacity to predict the presence of malignancy in B3-lesions has been investigated in various studies [30, 31].

**Fig. 9:**
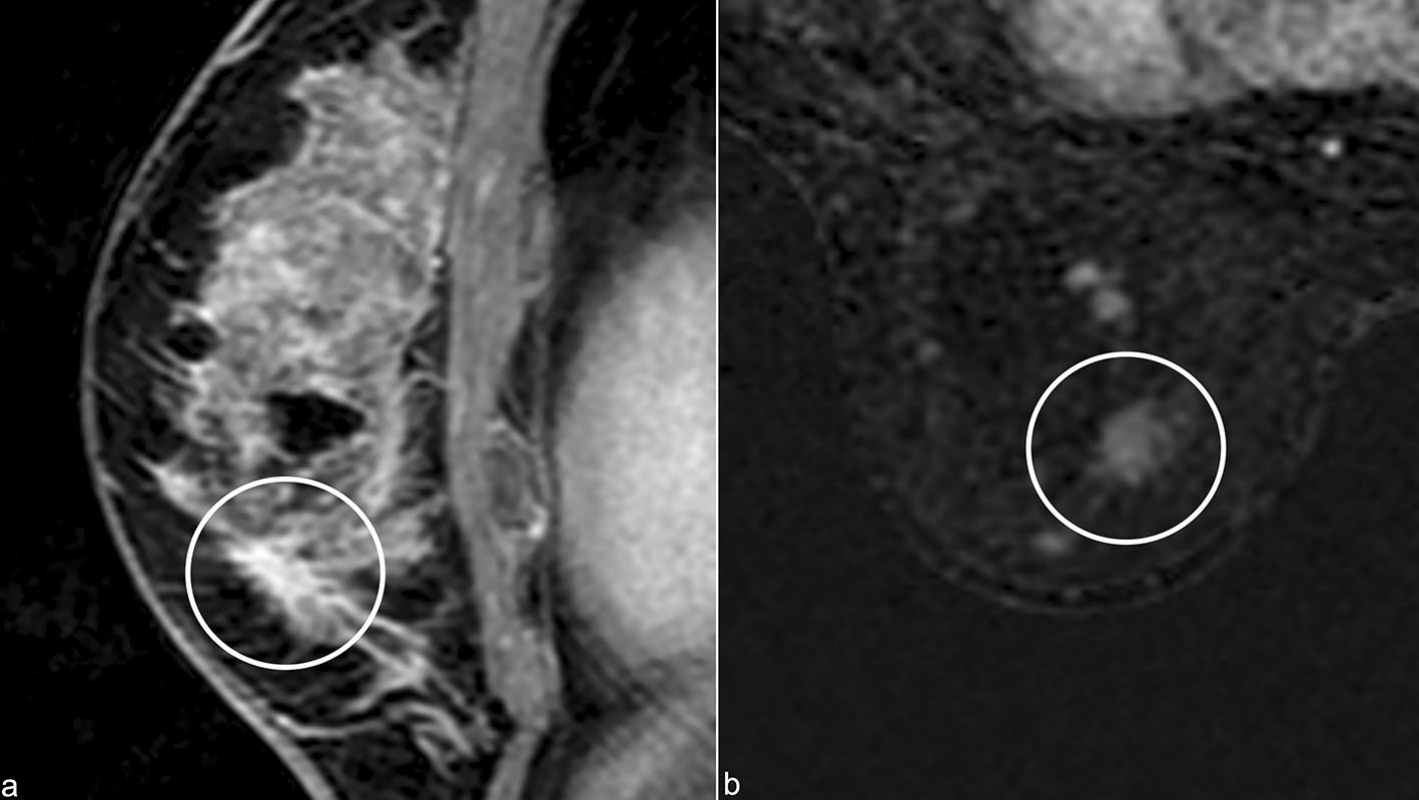
Sagittal MRI contrast material-enhanced T1-weighted image (**a**) and axial MRI subtracted early contrast-enhanced image (**b**) show an enhancing mass with irregular borders (white circle)

When RS is visible at MRI, three patterns of presentations have been identified, according to the literature [14]:

-Irregular or spiculated “tumor-like” mass. These lesions show the same morphology and enhancement kinetics of invasive breast cancer (Fig. 9);
-Stellate “architectural distortion,” without mass effect, with mild or no enhancement;
-Benign-looking oval or round mass with smooth margins and mild and gradual enhancement.

**Fig. 10:**
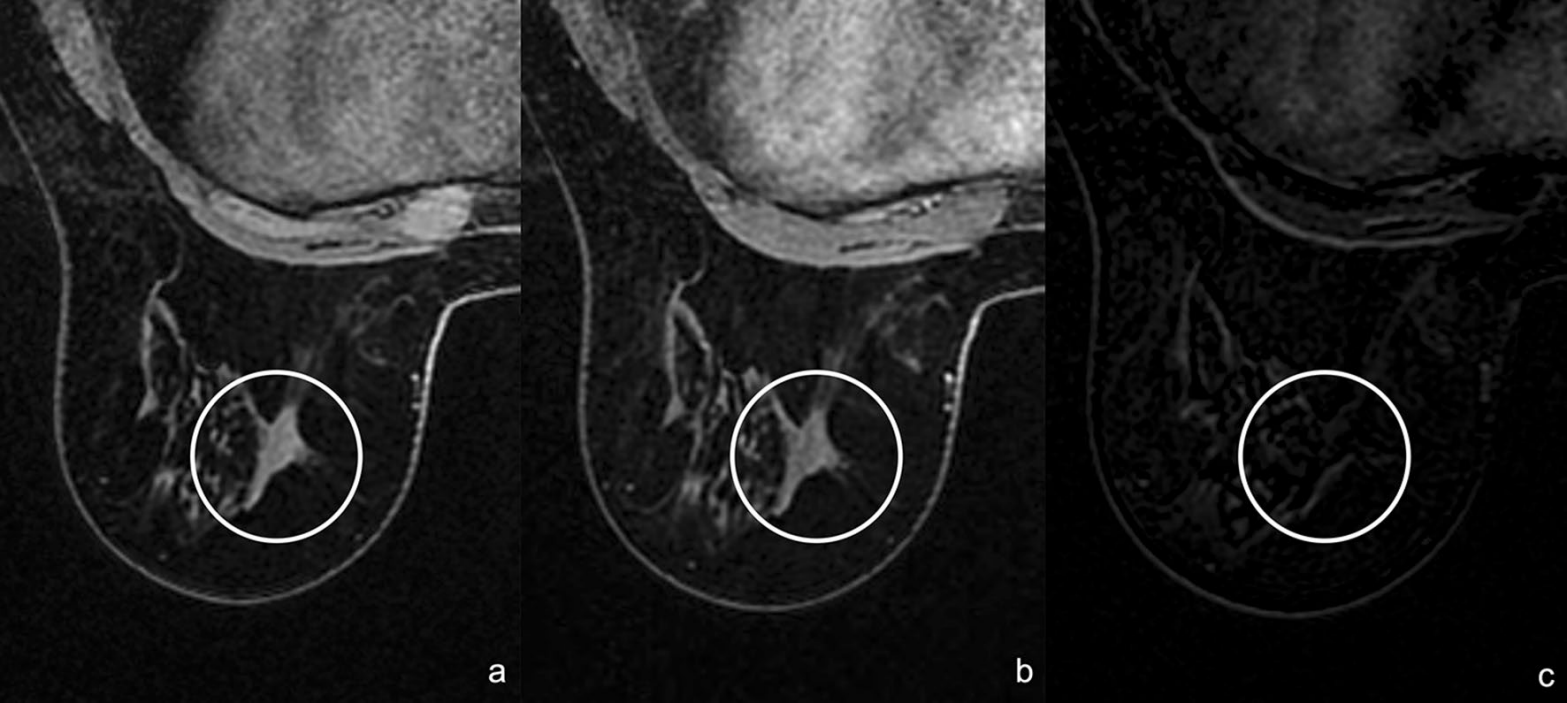
Axial MRI precontrast T1-weighted image (**a**), early contrast-enhanced T1-weighted image (**b**) and early T1-weighted subtraction show an architectural distortion without enhancement (white circle)

In some cases, RS could appear as an architectural distortion/mass without contrast enhancement (Fig. 10).

Several studies have investigated the role of MRI examinations in predicting the unfavorable evolution of lesions [32, 33], and a negative predictive value of 97.6–100% has been found for differentiating between benign and malignant RS lesions. These results suggest that in case of absent or modest enhancement, the possibility of malignancy can be excluded.

More recently, Santucci et al. [34], according to these statements, showed that upgrade was often associated with evident contrast-enhanced lesions. In contrast, Amitai et al. [11] found that MRI has low accuracy in differentiating invasive cancer from RS, with positive predictive value of 30%. In conclusion, in most of the cases, a clear-cut distinction of a RS versus invasive cancer is not possible. However, the role of MRI remains important to exclude the presence of other lesions either in the affected or in the contralateral breast; in fact, MRI detects many additional enhancing lesions unseen with DM and US [35].

## RS and malignancy

The rate of upgrade to carcinoma in RS’s excision specimens varies widely in the literature.

First autopsy studies provided an overall rate of malignancy of 8.6% (32/374 cases) in RS [6]. Afterward, upgrade rates diagnosed on CNB have ranged from 0 to 40% [36]. These studies were limited by the lack of radiological–pathological correlations, consistent criteria for excision, and clinical follow-up for patients who forego excision. Moreover, some papers highlighted the often eccentric and peripheral location of cancers present within RS locations that can conceivably evade a sampling needle [37].

There is a general agreement that RS alone is a benign lesion, but several studies in the literature acknowledge that the upgrade rate of RS depends on the presence or absence of associated atypia.

RS with no associated epithelial atypia has a very low rate of upgrade (< 10%) [38]. In fact, studies with carefully performed radiological–pathological correlations indicated that the upgrade rate for “RS without atypia” is 2% [39]. Moreover, several recent publications reported no cases upstaged to malignancy in the “RS without atypia” group [9]. Conversely, radial scars with atypia on biopsy show higher upgrade rates, with a range from 28% [40] to 36% [41] among the literature. A study of Ferreira et al. [42] found that the presence of atypia in the initial CNB was associated with an approximately 10 times higher risk for upstage at surgical excision**.**

In the last decade, the implementation of biopsy protocols using vacuum-assisted biopsy (VAB) with large needles (7–13 Gauge (G)) has permitted more extensive target lesion sampling at biopsy [43]. This has been associated with a progressive decline in the rate of underestimation of malignancy associated with the presence of RS alone on CNB [43]; in particular, a study of Linda et al. demonstrated that the biopsy underestimation rate of malignancy decreased from 9% for 14 G biopsies to 5% for 11 G biopsies [44].

The exact nature of the relationship between RS and neoplasia remains poorly understood.

Data from the Nurses’ Health Study suggested a stronger association between RS and hormone receptor-negative carcinomas [45] colliding with early clinical studies that reported RS to be most frequently found in conjunction with tubular carcinoma [46].

RS in some cases could be, indeed, misinterpreted as low-grade invasive ductal or tubular carcinoma. Eusebi et al. [47] addressed the distinctions between RS, infiltrating epitheliosis and tubular carcinoma. In most cases, hematoxylin and eosin and immunohistochemical staining for a surrounding layer of myoepithelial cells can differentiate RS from invasive cancer [48].

Further, gene mutations have been recently identified in the PIK3CA pathway in RS that are particularly prevalent in luminal-type, hormone receptor-positive breast cancers, offering additional insight into the pathogenesis of RS [49]. No consistent correlation has been demonstrated between malignancy at excision of RS and parity, menopausal status and clinical presentation. The only variable with a statistically significant relation to upgrading was the average age (> 50 years), associated with a slightly higher risk [50].

The most likely hypothesis is that the coexistence with high-risk lesions or the presence of breast tissue field, in addition to allowing development of RS may predispose tissue in the affected field to the development of carcinoma that is not etiologically related to RS [7, 51].

## Management

The management of RS is still debated. The majority of different academic institutions did not provide the same management recommendations for RS, this suggests that there is still a deep heterogeneity in the management of RS between breast imagers. RS represents approximately 0.09% of all CNB [52].

According to the recent International Consensus Conference, therapeutic excision with VAB or vacuum-assisted excision (VAE) is recommended after percutaneous diagnosis of RS [2], because of the potential sampling error due to the eccentric and peripheral location of invasive carcinoma present within RS, that can escape to a sampling needle [37, 53, 54]. In fact, adequate sampling of the periphery, as well as the center of the RS, improves the detection rate of associated atypia/malignancy [55]. In the past years, when a CNB returned a RS lesion, surgical excision was always suggested considering the potential sampling error. Nowadays, the aim of VAE is to take about 4 g of tissue, and the purpose is to equate a surgical biopsy, without the associated complications. The amount of tissue is estimated by multiplying the number of cores with estimated weight of each core dependent on the needle size [54, 56]; generally, a 7 or 8 G needle is used.

Thereafter, surveillance is justified [2].

Regarding surveillance, the National Health Service (NHS) Breast Screening multidisciplinary working group [38] recently suggested flowcharts for the management of RS, differentiating two pathways depending on the presence or the absence of atypia. In their opinion, VAE is always recommended in cases of RS with atypia, and if no additional atypia is found, surveillance with annual mammography is suggested. In cases where further atypia is found, the management should include open surgical excision [57]. In an interesting study of Özçağlayan et al. [58], breast lesion excision system (BLES) has been evaluated as a secure procedure that can provide high diagnostic success and serve as a therapeutic method in high-risk lesions, such as RS, with high complete excision rates.

The management is more controversial in cases without atypia. According to the NHS Breast Screening multidisciplinary working group [38], even in cases without atypia, VAE is always recommended, and the decision is based on the result obtained on histology; more specifically, if no atypia is retrieved after VAE, a three-yearly mammography is proposed. Some recent studies demonstrated that conservative management with imaging follow-up could be considered if the appropriate biopsy techniques are used and the pathology returns as isolated RS without atypia [38].

On a recent meta-analysis, Farshid et al. [59] focused on atypia and the extent of sampling as two potential factors to take into account for the substantial variation in reported upgrade rates. They observed that RS without atypia was the group of lesions with the lowest upgrade rate (1%) (95% CI 0–4%). Upgrade rates were significantly lower also for the group assessed by the 8-11G VAB than those by smaller biopsies. The authors concluded that imaging surveillance could be a reasonable option for RS without atypia assessed by 8-11G VAB. Likewise, Bacci et al. [60] found that VAB with a large core is reliable to exclude malignancy and allows avoiding surgical excision when there is no discordance between radiological and histological findings, and no associated atypia on biopsy. Eghtedari et al. [61] observed that in a group of 54 patients with a CNB histological result of RS without atypia, no case developed malignancy during the 2 years of follow-up (95% confidence interval 0–7%).

The recommendations could change according to lesion size. In a Slovenian study, Gašljević et al. [55] suggested that RS without atypia and smaller than 20 mm can be followed radiologically. Conversely, lesions larger than 20 mm, sampled with a smaller core and/or showing atypia, should be excised.

In addition, Linda et al. [14], as mentioned above, have demonstrated that MRI has a negative predictive value of 97.6% in evaluating malignant transformation in non-enhancing RS. Therefore, they concluded that an imaging follow-up could be suitable for non-enhancing RS, with a follow-up protocol of short interval MRI (every 6 months for 2 years) as a surveillance tool for patients with small RS without atypia on CNB.

Regarding suspicious lesions only MRI-visible, resulting to be a RS/CSL after MRI-guided biopsy, many studies in literature have evaluated the rates of upgrade to malignancy, showing a discordance. Some studies [62–64] revealed no upgrade to malignancy, others [65, 66] reported an overall upgrade to malignancy ranging from 15 to 23.1%. High upgrade rates could be explained by the lack of accuracy of the MRI biopsy technique. In fact, the number and dimension of samples may be responsible for the difference in upgrade rates. Ferreira et al. [42] indicated lower upgrade rates of RS with greater number of fragments obtained at biopsy and in RS subjected to VAB than in those subjected to core biopsy. In a recent study, Okamoto et al. [67] stated that when MRI biopsy is vacuum assisted, the risk of upgrade and malignancy is significantly lower with less indication for excisional biopsy.

It was tried to develop a predictive scoring system based on clinical–radiological–pathological data to choose the most appropriate management in US-detected B3 lesions [68]. The authors categorized RS as a “low-risk B3 lesion” and proposed a personalized strategy in every individual patient, considering the patient demographics, imaging features, and pathological results, with the objective of selecting the right management, reducing the frequency of benign surgical excision.

Grippo et al. [69] recently evaluated its feasibility based on clinical, pathological, and radiological data. It is assumed that a RS lesion with associated atypia should undergo therapeutic excision with VAB.

A multidisciplinary approach may be appropriate in patients with a diagnosis of RS without atypia, to decide on personalized management, which may include imaging surveillance or surgical excision based on patient risk factors, comorbid conditions, and their history of concurrent breast cancer.

In future studies, different imaging examinations features should be tested “in combination” to assess malignancy probability. Furthermore, emerging techniques, like radiomics (the extraction of tissue characteristics of tumor phenotype from images generating features not appreciated by the naked eye) and artificial intelligence, are showing promising results in evaluation of breast cancer [70] and in the future may provide additional information on the assessment of malignancy also in B3-lesions, integrating molecular and genetic findings.

## Conclusion

This review reported almost all the presentation patterns of RS through different imaging techniques already well-described among the literature and the updates on management.

It is important for the breast radiologist to be familiar with these features, in order to make an accurate differential diagnosis.

At present, no imaging examination can yet provide sufficient elements to certainly exclude malignancy. Despite this, all of them (DM/DBT, US, and MRI) provide a contribution in making the correct decision and, therefore, should all be performed. Moreover, biopsy is always recommended, and afterward, a systematic multidisciplinary evaluation is crucial. Besides, when the “wait-and-see” pathway is undertaken, it requires accurate and complete imaging examination protocols.

Additional studies including closer radiology–pathology correlations and development of artificial intelligence could help to reduce unnecessary excision biopsy and surgical procedures.

## Data Availability

Data sharing is not applicable to this article as no datasets were generated or analyzed.

## Abbreviations

ABUS: Automated Breast Ultrasound
BLES: Breast Lesion Excision System
CNB: Core Needle Biopsy
CSL: Complex Sclerosing Lesions
DBT: Digital Breast Tomosynthesis
DM: Digital Mammography
HHUS: Hand-Held Ultrasound
G: Gauge
MRI: Magnetic Resonance Imaging
NHS: National Health Service
RS: Radial Scar
US: Ultrasound
VAB: Vacuum-Assisted Biopsy
VAE: Vacuum-Assisted Excision

## References

[1] Ellis IO, Humphreys S, Michell M, Pinder SE, Wells CA, Zakhour HD, UK National Coordinating Commmittee for Breast Screening Pathology, European Commission Working Group on Breast Screening Pathology (2004). Best Practice No 179. Guidelines for breast needle core biopsy handling and reporting in breast screening assessment. J Clin Pathol.

[2] Rageth CJ, O’Flynn E, Pinker K, Kubik-Huch RA, Mundinger A, Decker T, Tausch C, Dammann F, Baltzer PA, Fallenberg EM, Foschini MP, Dellas S, Knauer M, Malhaire C, Sonnenschein M, Boos A, Morris E, Varga Z (2019) Second International Consensus Conference on lesions of uncertain malignant potential in the breast B3 lesions. Breast Cancer Res Treat 174(2):279–296 10.1007/s10549-018-05071-1 PMC6538569 30506111

[3] Loane J (2009). Benign sclerosing lesions of the breast. Diagn Histopathol.

[4] Wellings SR, Alpers CE (1984). Subgross pathologic features and incidence of radial scars in the breast. Hum Pathol.

[5] Battersby S, Anderson TJ (1985). Myofibroblast activity of radial scars. J Pathol.

[6] Nielsen M, Jensen J, Andersen JA (1985). An autopsy study of radial scar in the female breast. Histopathology.

[7] Cohen MA (2017). Radial scars of the breast encountered at core biopsy: review of histologic, imaging, and management considerations. Am J Roentgenol.

[8] Kennedy M, Masterson AV, Kerin M, Flanagan F (2003). Pathology and clinical relevance of radial scars: a review. J Clin Pathol.

[9] Martaindale S, Omofoye TS, Teichgraeber DC, Hess KR, Whitman GJ (2020). Imaging follow-up versus surgical excision for radial scars identified on tomosynthesis-guided core needle biopsy. Acad Radiol.

[10] Tabar L, Dean P (2001). Teaching atlas of mammography.

[11] Amitai Y, Scaranelo A, Menes TS, Fleming R, Kulkarni S, Ghai S, Freitas V (2020). Can breast MRI accurately exclude malignancy in mammographic architectural distortion?. Eur Radiol.

[12] Vijapura C, Yang L, Xiong J, Fajardo LL (2018). Imaging features of nonmalignant and malignant architectural distortion detected by tomosynthesis. Am J Roentgenol.

[13] Pujara AC, Hui J, Wang LC (2019). Architectural distortion in the era of digital breast tomosynthesis: outcomes and implications for management. Clin Imaging.

[14] Linda A, Zuiani C, Londero V, Cedolini C, Girometti R, Bazzocchi M (2012). Magnetic resonance imaging of radial sclerosing lesions (radial scars) of the breast. Eur J Radiol.

[15] Alleva DQ, Smetherman DH, Farr GH, Cederbom GJ (1999). Radial scar of the breast: radiologic-pathologic correlation in 22 cases. Radiographics.

[16] Frouge C, Tristant H, Guinebretiere J (1995). Mammographic lesions suggestive of radial scars: microscopic findings in 40 cases. Radiology.

[17] Mitnick JS, Vazquez MF, Harris MN, Roses DF (1989). Differentiation of radial scar from scirrhous carcinoma of the breast: mammographic-pathologic correlation. Radiology.

[18] Cherel P, Becette V, Hagay C (2005). Stellate images: anatomic and radiologic correlations. Eur J Radiol.

[19] Bouté V, Goyat I, Denoux Y, Lacroix J, Marie B, Michels J-J (2006). Are the criteria of Tabar and Dean still relevant to radial scar?. Eur J Radiol.

[20] Hagay C, Le Treut A, Dilhuydy MH (1988). Les images stellaires. Mammographie: guide d’interpre ´tation.

[21] Miller CL, West JA, Bettini AC (2014). Surgical excision of radial scars diagnosed by core biopsy may help predict future risk of breast cancer. Breast Cancer Res Treat.

[22] Partyka L, Lourenco AP, Mainiero MB (2014). Detection of mammographically occult architectural distortion on digital breast tomosynthesis screening: initial clinical experience. Am J Roentgenol.

[23] Ray KM, Turner E, Sickles EA, Joe BN (2015). Suspicious findings at digital breast tomosynthesis occult to conventional digital mammography: imaging features and pathology findings. Breast J.

[24] Bahl M, Lamb LR, Lehman CD (2017). Pathologic outcomes of architectural distortion on digital 2D Versus tomosynthesis mammography. Am J Roentgenol.

[25] Cawson JN (2005). Can sonography be used to help differentiate between radial scars and breast cancers?. Breast (Edinburgh, Scotland).

[26] Evans A, Whelehan P, Thomson K, McLean D, Brauer K, Purdie C, Jordan L, Baker L, Thompson A (2010). Quantitative shear wave ultrasound elastography: initial experience in solid breast masses. Breast Cancer Res.

[27] Zhi H, Ou B, Xiao XY, Peng YL, Wang Y, Liu LS, Xiao Y, Liu SJ, Wu CJ, Jiang YX, Parajuly SS, Xu P, Hao Y, Li J, Luo BM (2013). Ultrasound elastography of breast lesions in chinese women: a multicenter study in China. Clin Breast Cancer.

[28] Leong LC, Sim LS, Lee YS, Ng FC, Wan CM, Fook-Chong SM, Jara-Lazaro AR, Tan PH (2010). A prospective study to compare the diagnostic performance of breast elastography versus conventional breast ultrasound. Clin Radiol.

[29] Vourtsis A, Kachulis A (2018). The performance of 3D ABUS versus HHUS in the visualisation and BI-RADS characterisation of breast lesions in a large cohort of 1,886 women. Eur Radiol.

[30] Zuiani C, Londero V, Linda A, Girometti R, Bazzocchi M (2012). MRI in B3 lesions, low grade DCIS, high DCIS: is MR selecting the dangerous cases?. Eur J Radiol.

[31] Alsharif S, Aldis A, Subahi A, El Khoury M, Mesurolle B (2020). Breast MRI does not help differentiating radial scar with and without associated atypia or malignancy. Can Assoc Radiol J.

[32] Linda A, Zuiani C, Londero V, Bazzocchi M (2008). Outcome of initially only magnetic resonance mammography-detected findings with and without correlate at second-look sonography: distribution according to patient history of breast cancer and lesion size. Breast (Edinburgh, Scotland).

[33] Pediconi F, Occhiato R, Venditti F, Fraioli F, Napoli A, Votta V, Laghi A, Catalano C, Passariello R (2005). Radial scars of the breast: contrast-enhanced magnetic resonance mammography appearance. Breast J.

[34] Santucci D, Faiella E, Calabrese A (2019). Our radiological experience on b3 lesions: correlation between mammographic and MRI findings with histologic definitive result. Clin Breast Cancer.

[35] Mann RM, Balleyguier C, Baltzer PA, Bick U, Colin C, Cornford E, Evans A, Fallenberg E, Forrai G, Fuchsjäger MH, Gilbert FJ, Helbich TH, Heywang-Köbrunner SH, Camps-Herrero J, Kuhl CK, Martincich L, Pediconi F, Panizza P, Pina LJ, Pijnappel RM, European Society of Breast Imaging (EUSOBI), with language review by Europa Donna–The European Breast Cancer Coalition (2015). Breast MRI: EUSOBI recommendations for women’s information. Eur Radiol.

[36] Bianchi S, Caini S, Renne G, Cassano E, Ambrogetti D, Cattani MG, Saguatti G, Chiaramondia M, Bellotti E, Bottiglieri R, Ancona A, Piubello Q, Montemezzi S, Ficarra G, Mauri C, Zito FA, Ventrella V, Baccini P, Calabrese M, Palli D, VANCB Study Group (2011). Positive predictive value for malignancy on surgical excision of breast lesions of uncertain malignant potential (B3) diagnosed by stereotactic vacuum-assisted needle core biopsy (VANCB): a large multi-institutional study in Italy. Breast (Edinburgh, Scotland).

[37] López-Medina A, Cintora E, Múgica B, Operé E, Vela AC, Ibañez T (2006). Radial scars diagnosed at stereotactic core-needle biopsy: surgical biopsy findings. Eur Radiol.

[38] Pinder SE, Shaaban A, Deb R, Desai A, Gandhi A, Lee A, Pain S, Wilkinson L, Sharma N (2018). NHS Breast Screening multidisciplinary working group guidelines for the diagnosis and management of breast lesions of uncertain malignant potential on core biopsy (B3 lesions). Clin Radiol.

[39] Chou W, Veis DJ, Aft R (2018). Radial scar on image-guided breast biopsy: is surgical excision necessary?. Breast Cancer Res Treat.

[40] Brenner RJ, Jackman RJ, Parker SH (2002). Percutaneouscoreneedlebiopsy of radial scars of the breast: when is excision necessary?. Am J Roentgenol.

[41] Rakha EA, Lee AH, Jenkins JA (2011). Characterization and outcome of breast needle core biopsy diagnoses of lesions of uncertain malignant potential (B3) in abnormalities detected by mammographic screening. Int J Canc.

[42] Ferreira AI, Borges S, Sousa A, Ribeiro C, Mesquita A, Martins PC, Peyroteo M, Coimbra N, Leal C, Reis P, Sousa JA (2017). Radial scar of the breast: Is it possible to avoid surgery?. Eur J Surg Oncol.

[43] Conlon N, D'Arcy C, Kaplan JB, Bowser ZL, Cordero A, Brogi E, Corben AD (2015). Radial scar at image-guided needle biopsy: is excision necessary?. Am J Surg Pathol.

[44] Linda A, Zuiani C, Furlan A (2010). Radial scars without atypia diagnosed at imaging-guided needle biopsy: how often is associated malignancy found at subsequent surgical excision, and do mammography and sonography predict which lesions are malignant?. Am J Roentgenol.

[45] Aroner SA, Collins LC, Connolly JL, Colditz GA, Schnitt SJ, Rosner BA, Hankinson SE, Tamimi RM (2013). Radial scars and subsequent breast cancer risk: results from the Nurses' Health Studies. Breast Cancer Res Treat.

[46] Vega A, Garijo F (1993). Radial scar and tubular carcinoma Mammographic and sonographic findings. Acta radiologica (Stockholm, Sweden: 1987).

[47] Eusebi V, Millis RR (2010). Epitheliosis, infiltrating epitheliosis, and radial scar. Semin Diagn Pathol.

[48] Calhoun BC (2018). Core needle biopsy of the breast: an evaluation of contemporary data. Surgical Pathol Clin.

[49] Wolters KL, Ang D, Warrick A, Beadling C, Corless CL, Troxell ML (2013). Frequent PIK3CA mutations in radial scars. Diagn Mol Pathol.

[50] Ha SM, Cha JH, Shin HJ, Chae EY, Choi WJ, Kim HH, Oh HY (2018). Radial scars/complex sclerosing lesions of the breast: radiologic and clinicopathologic correlation. BMC Med Imaging.

[51] Falomo E, Adejumo C, Carson KA, Harvey S, Mullen L, Myers K (2019). Variability in the management recommendations given for high-risk breast lesions detected on image-guided core needle biopsy at U.S. academic institutions. Curr Prob Diagn Radiol.

[52] Boateng S, Tirada N, Khorjekar G, Richards S, Ioffe O (2020). Excision or observation: the dilemma of managing high-risk breast lesions. Curr Probl Diagn Radiol.

[53] Alvarado-Cabrero I, Tavassoli FA (2000). Neoplastic and malignant lesions involving or arising in a radial scar: a clinicopathologic analysis of 17 cases. Breast J.

[54] NHS Breast Screening Programme (2016) Clinical guidance for breast cancer screening assessment. NHSBSP publication no. 49. 4th edn

[55] Gašljević G, Hertl K, Gazić B, Lamovec J, Žgajnar J (2020). Reducing indications for radial scar surgical excision in Slovenian breast cancer screening program. Ann Diagn Pathol.

[56] O'Flynn EA, Wilson AR, Michell MJ (2010). Image-guided breast biopsy: state-of-the-art. Clin Radiol.

[57] Rakha E, Beca F, D'Andrea M, Abbas A, Petrou-Nunn W, Shaaban AM, Kandiyil A, Smith S, Menon S, Elsheikh S, ElSayed ME, Lee AH, Sharma N (2019). Outcome of radial scar/complex sclerosing lesion associated with epithelial proliferations with atypia diagnosed on breast core biopsy: results from a multicentric UK-based study. J Clin Pathol.

[58] Kurtoğlu Özçağlayan Tİ, Özkan Gürdal S, Öznur M, Özçağlayan Ö, Doğru M, Topçu B (2019). Effectiveness of the diagnostic pathway of BLES: could it be safely used as a therapeutic method in selected benign lesions?. Diagn Interventional Radiol (Ankara, Turkey).

[59] Farshid G, Buckley E (2019). Meta-analysis of upgrade rates in 3163 radial scars excised after needle core biopsy diagnosis. Breast Cancer Res Treat.

[60] Bacci J, MacGrogan G, Alran L, Labrot-Hurtevent G (2019). Management of radial scars/complex sclerosing lesions of the breast diagnosed on vacuum-assisted large-core biopsy: is surgery always necessary?. Histopathology.

[61] Eghtedari M, Le-Petross H, Babiera GV, Albarracin CT, Hess KR, Woodtichartpreecha P, Whitman GJ (2019). Not all patients with a diagnosis of a radial scar require excision. Breast J.

[62] Strigel RM, Eby PR, Demartini WB, Gutierrez RL, Allison KH, Peacock S, Lehman CD (2010). Frequency, upgrade rates, and characteristics of high-risk lesions initially identified with breast MRI. Am J Roentgenol.

[63] Crystal P, Sadaf A, Bukhanov K, McCready D, O'Malley F, Helbich TH (2011). High-risk lesions diagnosed at MRI-guided vacuum-assisted breast biopsy: can underestimation be predicted?. Eur Radiol.

[64] Speer ME, Huang ML, Dogan BE, Adrada BE, Candelaria RP, Hess KR, Hansakul P, Yang WT, Rauch GM (2018). High risk breast lesions identified on MRI-guided vacuum-assisted needle biopsy: outcome of surgical excision and imaging follow-up. Br J Radiol.

[65] Lourenco AP, Khalil H, Sanford M, Donegan L (2014). High-risk lesions at MRI-guided breast biopsy: frequency and rate of underestimation. Am J Roentgenol.

[66] Heller SL, Elias K, Gupta A, Greenwood HI, Mercado CL, Moy L (2014). Outcome of high-risk lesions at MRI-guided 9-gauge vacuum- assisted breast biopsy. Am J Roentgenol.

[67] Okamoto S, Chen ST, Covelli JD, DeMartini WB, Daniel BL, Ikeda DM (2020). High-risk lesions diagnosed at MRI-guided vacuum-assisted breast biopsy: imaging characteristics, outcome of surgical excision or imaging follow-up. Breast Cancer (Tokyo, Japan).

[68] Giuliani M, Rinaldi P, Rella R, D'Angelo A, Carlino G, Infante A, Romani M, Bufi E, Belli P, Manfredi R (2018). A new risk stratification score for the management of ultrasound-detected B3 breast lesions. Breast J.

[69] Grippo C, Jagmohan P, Clauser P, Kapetas P, Meier A, Stöger AM, D'Angelo A, Baltzer P (2020). External validation of a risk stratification score for b3 breast lesions detected at ultrasound core needle biopsy. Diagnostics (Basel, Switzerland).

[70] Lee SH, Park H, Ko ES (2020). Radiomics in breast imaging from techniques to clinical applications: a review. Korean J Radiol.

